# Online Patient Education Resources for Anterior Cruciate Ligament Reconstruction: An Assessment of the Accuracy and Reliability of Information on the Internet Over the Past Decade

**DOI:** 10.7759/cureus.46599

**Published:** 2023-10-06

**Authors:** Alvarho J Guzman, Therese Dela Rueda, Nicholas Williams, Shane Rayos Del Sol, Sarah Jenkins, Caleb Shin, Stewart Bryant, Patrick McGahan, James Chen, MD, MPH

**Affiliations:** 1 Orthopedic Surgery, Advanced Orthopedics & Sports Medicine, San Francisco, USA; 2 Orthopedic Surgery, Albany Medical College, Albany, USA; 3 Orthopedic Surgery, University of Connecticut School of Medicine, Farmington, USA

**Keywords:** orthopedic sports medicine, acl tear, anterior cruciate ligament (acl), anterior cruciate ligament (acl) reconstruction, ortho surgery

## Abstract

Purpose: The purpose of this study is to evaluate the quality of patient education materials accessible through popular online search engines regarding anterior cruciate ligament (ACL) injuries and anterior cruciate ligament reconstruction (ACLR).

Methods: Two search terms (“ACL surgery” and “ACL reconstruction”) were entered into three search engines (Google, Yahoo, and Bing). The quality of information was scored using a novel scoring system developed and overseen by sports medicine orthopedic clinical research fellows and fellowship-trained orthopedic surgeons. Website quality, credibility, and readability were further assessed by the DISCERN score, *Journal of the American Medical Association *(JAMA) benchmark criteria, and Flesch-Kincaid Reading Grade Level (FKRGL), respectively. The Health On the Net Code of Conduct (HONcode) certification was also utilized to assess the transparency of health information for each website.

Results: We evaluated 39 websites. The average score for all websites was 11.2±5.6 out of 28 total points. Six out of the 39 websites (41%) were HONcode certified. The websites that contained HONcode certification had a higher average JAMA benchmark score (3.5±0.7) and DISCERN score (44.6±14.7) when compared to the websites without the certification, 2.2±1.2 and 37.6 ± 15.9 for JAMA and DISCERN, respectively. The mean JAMA benchmark score was 2.7±1.2 (67.5%) for all websites out of a possible four points. The average FKRGL for all 39 websites was 10.0±2.0 (range: 5.4-13).

Conclusion: The quality of patient education materials accessible on the internet regarding ACL injuries and ACLR can be misleading and directly impact the patient's decision-making process essential to the patient-physician relationship over the past decade.

Clinical Relevance: The internet can be a helpful online resource, however, surgeon clarification and consultation with qualified healthcare professionals are strongly recommended prior to clinical decision-making regarding potential treatment options.

## Introduction

An anterior cruciate ligament (ACL) injury is one of the most common knee injuries in orthopedics with an annual incidence of 68.6 per 100,000 person-years in the general population [1]. In the United States, ACL tears comprise 50% of knee injuries with more than 200,000 people affected annually [2]. There are an estimated 80,000 to 100,000 anterior cruciate ligament reconstructions (ACLR) in the United States each year [3]. Moreover, a 2016 population-based study by Sanders et al. conducted between 1990 and 2010 estimated that the rate of reconstruction has been steadily increasing, estimating that about 75% of adults undergo reconstruction within the first year of injury [1]. Given the increased frequency of ACL reconstructions and the overall lack of studies examining patient satisfaction, patient education may allow patients to be better informed prior to surgery, giving them an environment for shared decision-making [3-5]. An emphasis on patient education can also reduce any apprehension toward possible risks associated with surgery and the demands of the rehabilitation process [4-7]. Furthermore, a 2021 study by Nwachuku et al. analyzed patient-reported outcomes at medium to long-term follow-up and found that patient satisfaction and return to play after ACL reconstruction are high, but more robust quality studies examining patient satisfaction are needed to understand the overall value of ACLR [8]. 

As of July 2020, approximately 312 million Americans had internet access totaling 95.6% of the overall population [9]. Given the overall availability of the internet, patients can use the internet as a major educational tool regarding certain medical conditions, procedures, treatment, and prognosis [4,5,10]. The Internet is readily accessible for most patients and provides a time-efficient and cost-effective way to take an active role in their care, resulting in the improvement of self-care and social support [11]. Thus, the internet can offer physicians and patients a vast amount of medical material instantaneously, at any time, from anywhere in the world. Numerous studies that examined the quality of educational materials from search engine results have often concluded major variability in the reliability and quality of information readily available [12-16]. Regarding ACL injuries and reconstruction, a 2013 study by Bruce-Brand et al. found that many online resources lacked basic information such as prognosis and treatment options [4]. Similarly, in 2013 Duncan et al. reported the quality of information available on search engines regarding ACLR appears mixed and many websites underreport important factors such as patient eligibility and potential complications [5]. Although the benefits of a patients’ active involvement in their personal care are well established, the overall poor quality of online search engine resources had the risk of causing further uncertainty and health anxiety [17].

The purpose of this study is to evaluate the quality of patient education materials regarding ACLR over the past decade that are easily accessible through the most popular online search engines. In contrast to previous 2013 studies by Bruce-Brand et al. and Duncan et al. analyzing accessible ACLR patient education materials, our study incorporates how trending in search engine results have changed and improved over the last 10 years. Using a novel scoring system created by the authors and finalized by a sports medicine-certified orthopedic surgeon, we determine the accuracy with which each website addresses the etiology, preoperative evaluation, treatment options, risks, and prognosis of ACL tears and reconstruction. Furthermore, this study presents a proper 10-year update with a variety of scoring systems that examine a website's overall objectivity, accuracy, quality, and readability.

## Materials and methods

Google, Yahoo, and Bing were the three search engines used given their overall popularity for search. The search terms “ACL surgery” and “ACL reconstruction” were used. The first 25 results for each term were collected in each of the search engines, which resulted in a total of 6 searches (two search terms × three search engines. A total of 150 websites were recorded, and this search was completed in September 2020.

All websites were initially organized by search terms and search engines. Duplicate websites were noted and excluded. Additional websites that were excluded were those that were blogs/testimonials, broken, irrelevant toward ACL reconstruction, solely focused on the cost of an ACL reconstruction, selling a specific product, peer-reviewed primary literature, video content, physician-specific videos, and medical professional-specific content. Physician-specific videos and content for medical professionals were excluded because of their intended audience, and they contained content beyond the scope of patient education. In addition, websites that mainly featured a video for education were excluded since the grading tools (i.e., DISCERN) used to grade the overall quality of websites were not intended for the evaluation of videos. Each website was categorized as academic, allied non-physician, physician, commercial, and unspecified.

Once a list of websites was established, the list of websites and a scoring rubric were provided to three different independent evaluators. Two of the three graders were orthopedic clinical research fellows in sports medicine. The scoring rubric created is author-derived with oversight from a sports medicine fellowship-trained orthopedic surgeon. The components of the scoring rubric are based on UpToDate, the American Academy of Orthopaedic Surgeons (AAOS), and the textbook “ACL injury and its Treatment” by Mitsuo Ochi. The rubric was initially created by the three evaluators, and it was ultimately agreed upon by the sports medicine orthopedic surgeon. Each component is categorized into preoperative, surgical, and postoperative/prognosis components. Each component included were those that were deemed essential for patient education. Scoring components included the function of the ACL, symptoms and evaluation of an ACL rupture, treatments, benefits and risks, and prognosis, and each criterion was valued as one point. Once all the websites were scored, all the values were collected and the mean was calculated. 

Since the author-derived rubric is based on whether a website mentions a specific topic, the DISCERN was used to evaluate overall quality, accuracy, and bias. DISCERN is a commonly used scoring system used to grade written consumer health information regarding the treatment options for patients. Each of the 16 questions in the DISCERN is graded from one to five based on how effectively a website addresses each category. The DISCERN score examines relevance, use of peer-reviewed literature, discussion of treatment options, risks and benefits for treatments, prognosis, and if the patient plays an active role in determining treatment. 

The JAMA criteria score was used to evaluate the credibility of the paper. These criteria specifically examined authorship, attribution, disclosure, and currency. Authorship requires the authors and contributors to provide their affiliations. Attribution refers to the listing of references and sources as well as copyright information. Disclosure assesses whether website ownership, conflict of interest, sponsorship, advertising, commercial funding, or conflict of interests is fully disclosed. Currency requires that each website provide accurate dates when content is posted and updated. One point is added for each criterion met, with a maximum of four points, a score of four indicates a credible source and a score of zero indicates unreliable information.

The Flesch-Kincaid Reading Grade Level (FKRGL) examined the readability of a website and assessed the difficulty in which information from each website can be understood and comprehended. FKGL indicates the academic grade level required to understand written material, determined by a formula that considers the number of words per sentence and the number of syllables per word. Scores range from 0 to 18, with 0 being a level of just learning to read, a kindergarten level, and 18 being a level on par with an academic paper, a college readability level.

Health On the Net Code of Conduct (HONcode) certification is an ethical standard utilized as a mark of certification for medical and health websites to publish transparent health-related information. The presence or absence of HONcode certification is listed for each website.

## Results

**Figure 1:**
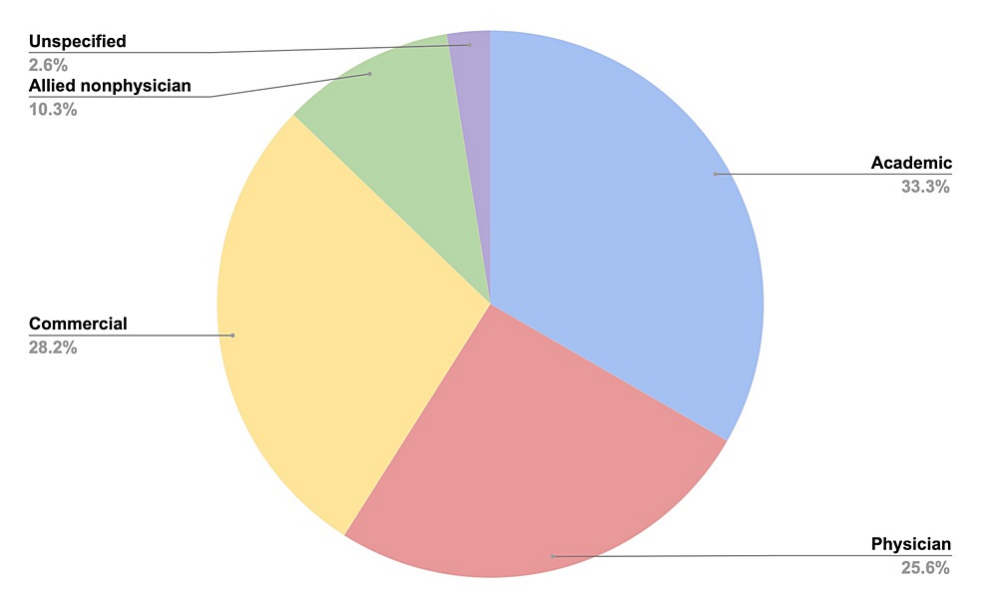
Percent Distribution of Website by Topic

After filtering through the initial 150 websites found, a total of 39 websites that addressed ACL surgery and reconstruction were included. Figure 1 shows the overall percent distribution of websites by topic. The majority of websites used were academic (33.3%). A summary of the website scores by each grading criteria is highlighted in Table 1.

**Table 1:** Summary of Website Scores HONcode: Health On the Net Code of Conduct certification.

		ACL Scoring		JAMA Score		DISCERN		FK	
Type of Site	(n)	Mean	SD	Mean	SD	Mean	SD	Mean	SD
Overall	39	11.2	5.6	2.7	1.2	41.3	15.5	10.0	2.0
HONcode (yes)	16	10.8	5.3	3.5	0.7	44.6	14.7	9.9	1.7
HONcode (no)	23	11.6	5.9	2.2	1.2	37.6	15.9	10.2	2.2
Academic	13	12.9	6.3	2.2	1.4	44.3	15.9	9.4	2.1
Physician	10	9.5	5.3	1.8	1.2	29.1	10.2	10.0	3.6
Commercial	11	10.0	5.6	3.4	1.2	44.4	16.6	9.7	1.8
Allied nonphysician	4	12.3	5.3	2.4	1.8	43.3	17.0	7.8	4.9
Unspecified	1	12.7	4.7	-	-	56.7	11.8	-	-

Overall, the included 39 websites had an average score of 11.2±5.6 out of 28 total points (Table 1). The criteria with the highest percentage representation from all articles were “physical therapy (76%)” and “rehab protocol (76%).” The criteria with the lowest percentage representation were “role in proprioception (9%)” and “single vs. double vs. triple bundle repair (10%).” By subsection, the websites scored an average of 3.19±2.75 (31.9%) out of 10 total points for the presurgical category, 3.69±2.14 (41%) out of nine total points for the surgical category, and 4.14±2.03 (51.8%) out of eight total points for the postsurgical category (Table 2). Table 3 shows the top five highest-scored websites, scoring an average of 18.3 vs. 10.3 for all the remaining websites.

**Table 2:** Average Website Scores by Topic ACL, anterior cruciate ligament; AP, anterior to posterior; MRI, magnetic resonance imaging

Percent of Websites Including Topic	
Pre-surgical	Website Percentage (%)
Biochemical function of ACL	50
Role in proprioception	9
Age	33
Sex	20
Mechanism of Injury	50
Meniscal tear associated with ACL tear	33
Grade 1 vs. 2 vs. 3 sprain	18
Symptoms/functional disability (instability, reduced ROM, tenderness over joint line)	52
Plain radiographs (AP, lateral)	25
MRI	30
Surgical	Website Percentage (%)
Reconstruction: patellar tendon	68
Reconstruction: hamstring tendon	74
Reconstruction: allograft	74
Single vs. double vs. triple bundle repair	10
Debridement +/- partial meniscectomy	20
Anatomic reconstruction	28
Revision reconstruction	10
Intraoperative complications	38
Expected outcomes and success rates of surgery	51
Treatment/postoperative	Website Percentage (%)
Rest and bracing	57
Physical therapy	76
Return to sport/activity	74
Rehab protocol	76
Continued instability	26
Risk of infection	50
Graft failure	41
ACL prevention	21

**Table 3:** Top Five Websites

Top Five Websites	Mean Quality
https://orthoinfo.aaos.org/en/treatment/acl-injury-does-it-require-surgery/	18.3
https://www.emedicinehealth.com/torn_acl/article_em.htm	18.3
https://www.sports-health.com/sports-injuries/knee-injuries/acl-tear-surgical-repair	18.3
https://www.verywellhealth.com/acl-surgery-making-a-decision-2548473	18.3
https://my.clevelandclinic.org/health/diseases/16576-anterior-cruciate-ligament-acl-injuries-	18.3

Table 1 shows the relationship between outcome scores and HONcode certification. Sixteen out of the 39 websites (41%) were HONcode certified. The websites that contained HONcode certification had a higher average JAMA benchmark score (3.5±0.7) and DISCERN score (44.6±14.7) when compared to the websites without the certification, 2.2±1.2 and 37.6±15.9 for JAMA and DISCERN, respectively.

The mean JAMA benchmark score was 2.7±1.2 (67.5%) for all websites out of a possible four points (Table 1). The commercial (3.4) and allied nonphysician (2.4) categorized sites scored the highest averages.

The average FKRGL for all 39 websites was 10.0±2.0 (range: 5.4-13). A FKRGL of 10 indicates readability at a ninth-grade level.

## Discussion

ACL injuries are among the most common knee injuries in orthopedics and the general population with the incidence of ACLR steadily increasing [18]. Given the current ease of access to internet availability, the internet can be a reliable, cost-effective, and efficient model for public dissemination of medical information [19-21]. Several studies analyzing the quality of available medical information online that patients encounter, particularly in relation to orthopedics and sports medicine, have been of mixed quality and do not provide the most effective resources [22-27]. Substandard information available online has the potential to impact the patient-physician relationship and negatively influence patients' expectations regarding diagnosis, prognosis, and treatment options [28]. Furthermore, a 2021 review by Schwarz et al. regarding internet-based information on orthopedic sports medicine emphasized the active role physicians must play in improving the quality of online information and increasing the health literacy of their patients [29]. 

This study included 39 total websites with publicly available information regarding ACL surgery and ACLR found through the popular search engines Google, Yahoo, and Bing. The criteria with the highest percentage representation from all articles were “physical therapy (76%)” and “rehab protocol (76%)" and the criteria with the lowest percentage representation were “role in proprioception (9%)” and “single vs. double vs. triple bundle repair (10%).” The five highest scored websites scored an average of 18.3 points vs. 10.3 for all remaining websites. Therefore, we conclude the impact of information on certain websites is variable and more emphasized in some versus others.

HONcode certification was evaluated to assess the relationship between reliability, credibility, and transparency in relation to its quality. Sixteen out of the 39 websites were HONcode certified (41%) and websites that contained HONcode certification had a higher average JAMA benchmark score (3.5±0.7) and DISCERN score (44.6±14.7) when compared to the websites without the certification, (2.2±1.2) and (37.6±15.9) for JAMA and DISCERN, respectively. Thus, our study indicates that HONcode certification reliably identifies websites with higher quality and content scores which previous studies have also identified across various specialties in medicine [30-31]. Concerning topics in orthopedics and sports medicine, Starman et al. found the content and quality of publicly available health information is highly variable, and HONcode certification is a reliable marker of websites with compliance and transparency [12].

The mean JAMA benchmark score was 2.7±1.2 (67.5%) for all websites out of a possible four points. This JAMA score is relatively higher than other scores from previous orthopedic studies evaluating available online medical information [4,32-35]. We can attribute this relatively higher average JAMA benchmark score to this study being an updated study assessing the quality and content of available online medical information, in addition to the fact we took into account the importance of trending in search engines. However, we did not find a significant relationship between the JAMA benchmark score and internet quality, thus credibility established by the JAMA benchmark score does not necessarily signify enhanced quality of information for a given website, a finding also reported by Goldenberg et al. [27].

The majority of websites in this study were academic (33.3%) with an average score of 11.2±5.6 out of 28 total points. Consistent with previous studies, we report websites of an academic nature provide better information quality, and patients searching on the internet can reliably utilize academic institutions for educational resources [36-37]. Moreover, a 2021 study by Wally et al. assessing the quality and content of orthopedic conditions addressing older adults found high variability in available information addressing topics such as osteoporosis and fragility fractures and ultimately encouraged patients to access reputable sites that are academically driven [36]. The average FKRGL for all 39 websites was 10.0±2.0 (range: 5.4-13). Given that our lowest-scored article was ranked at the fifth-grade level (5.4%) and our highest-scored article was ranked at the college reading level (13%), this finding shows considerable variability of medical information online on an academic level accessible to patients. A 2021 study by Kruse et al. evaluating the readability, content, and quality of COVID-19 education materials online found that although a wide availability of patient education materials is available online, readability is significantly higher than the recommended sixth-grade reading level advised by the National Institute of Health and US Department of Health [37]. Therefore, the internet has a critical role in providing readable patient education materials on a large, accessible platform so the general population can be accurately informed on medical information that can potentially improve overall health outcomes.

Limitations

There are several limitations to this study we must address to establish full transparency and objectivity of our presented data. A weakness of this study is the relative subjective nature in which each website was graded. Moreover, our study results are only applicable insofar as the search terms “ACL surgery” and “ACL reconstruction” are used. Although we have developed a novel scoring system that took into account how trending in search engines have changed or improved over time, our scoring system does analyze similar criteria used in previous ACLR patient information studies such as the JAMA benchmark criteria, DISCERN score, and FKRGL. We have also subjectively assumed which websites provide the most reliable and transparent information for a given search engine, which is accessible to the patient. This does not take into account the patient's individual factors that can limit their ability and capacity to access health-related information on the Internet. Furthermore, current internet trends and advances in search engine technology are constantly changing so our presented data may not accurately reflect the most up-to-date health information regarding ACLR available online.

## Conclusions

Patient education materials available on the web regarding ACL injuries and ACLR through popular search engines such as Google, Yahoo, and Bing can provide the patient with useful resources but may be of variable reliability. After a thorough analysis of available information on the web, while incorporating how trending in search engines have improved and changed over the last 10 years, we conclude that the internet can help differentiate potential treatment options but surgeon clarification in unison with a healthcare professional team is strongly recommended before any clinical decision-making.
